# Measuring Person- and Task-Centered Dementia Mealtime Care: Validating Clinically Practical Tools

**DOI:** 10.1093/geroni/igaf122.1572

**Published:** 2025-12-31

**Authors:** Wen Liu, Yelena Perkhounkova, Maria Hein

**Affiliations:** Penn State University, University Park, Pennsylvania, United States; University of Iowa, Iowa City, Iowa, United States; University of Iowa, Iowa City, Iowa, United States

## Abstract

Person-centered mealtime care (PCMC) is assistance prioritizing accommodations of individual abilities, needs, and preferences and engaging individuals in eating through dyadic interactions and environmental stimulations. Task-centered mealtime care (TCMC) is assistance prioritizing completion of eating activities without adequate considerations of individual needs and preferences. Maximizing PCMC and minimizing TCMC is critical to optimize mealtime care quality and outcomes for people with dementia. Few tools are valid and clinically practical to measure PCMC and TCMC. We adapted the Cue Utilization and Engagement in Dementia (CUED) video-coding scheme, a novel and valid but resource-intensive tool, into two observational scales and examined their structural validity to assess PCMC and TCMC. 424 videos of dyadic mealtime interactions (mean duration=25.8 minutes, range=1-142 minutes) were coded using CUED, capturing 66 staff (age mean=39.6 years; 84.9% female; 22.7% non-white; 16.9% Hispanic) in 10 nursing homes. Staff behaviors were categorized based on frequency: 0 (not observed), 1 (observed 1-2 times), and 2 (observed ≥3 times). Several behaviors were combined based on content relevance and redundancy, resulting in 16 PCMC items and 5 TCMC items. For PCMC, 20 random splits of videos were created for exploratory (EFA) and confirmatory factor analyses (CFA): 1- and 2-factor structures with 8-9 items were suggested by EFA; an 8-item 1-factor scale (Cronbach alpha=.75) was chosen based on conceptual interpretability, CFA model fit, and internal consistency. A 3-item TCMC scale (Cronbach alpha=.32) was identified. Future testing is needed using the scales for direct observations of mealtime care in nursing homes to establish clinical utility.

